# 2899. Integrating HIV Partner Services and Molecular Epidemiology Data to Enhance HIV Transmission Disruption in Rhode Island: Findings from a Public Health-Academic Partnership

**DOI:** 10.1093/ofid/ofad500.170

**Published:** 2023-11-27

**Authors:** Aditya Khanna, Vladimir Novitsky, August Guang, Mark Howison, Fizza S Gillani, Jon Steingrimsson, Casey Dunn, John Fulton, Thomas Bertrand, Katharine Howe, Lila Bhattarai, Guillermo Ronquillo, Meghan MacAskill, Utpala Bandy, Rami Kantor

**Affiliations:** Brown University, Providence, RI; Alpert Medical School of Brown University, Providence, Rhode Island; Brown University, Providence, RI; Research Improving Peopl'es Lives, Providence, Rhode Island; Brown Alpert Medical School, Providence RI, USA, Providence, Rhode Island; Brown University, Providence, RI; Yale University, New Haven, Connecticut; Brown University, Providence, RI; Rhode Island Department of Health, Providence, Rhode Island; Rhode Island Department of Health, Providence, Rhode Island; Rhode Island Department of Health, Providence, Rhode Island; Rhode Island Department of Health, Providence, Rhode Island; Rhode Island Department of Health, Providence, Rhode Island; Rhode Island Department of Health, Providence, Rhode Island; Alpert Medical School of Brown University, Providence, Rhode Island

## Abstract

**Background:**

HIV remains a significant public health concern. Both contact tracing (identifying and notifying partners of people diagnosed with HIV) and molecular epidemiology (phylogenetic inference and cluster detection), are used to disrupt transmission. Integration of both modalities may be synergistic, though it is not evaluated or implemented routinely.

**Methods:**

To assess whether integrating HIV contact tracing and molecular epidemiology data are more informative for public health than using each separately, we evaluated concordance between a statewide public health contact tracing database (CTDB) collected in 2008-2021 and handled by the Rhode Island Department of Health, and a statewide individual-level HIV-1 *pol* sequence database (SDB) collected in 2004-2021 by Brown University investigators. Molecular clusters were identified using at least one of seven common phylogenetic methods. Concordance (overlap in persons appearing in both databases) was evaluated using the Jaccard Similarity Coefficient (JSC).

**Results:**

The CTDB included disease intervention specialist interview data from 2469 unique persons (2468 newly diagnosed; 1458 named partners) and the SDB included sequences from 3266 persons. There were 920 persons who appeared in both databases, while 2346/3266 (72%) appeared in the SDB but not CTDB, and 1549/2469 (63%) appeared in the CTDB but not SDB. Of the 351 molecular clusters identified, 156 (44%) consisted of persons also in the CTDB. The JSC between the SDB and CTDB was 0.19. Of the 920 persons in both databases, 509 newly diagnosed persons provided partner data and 63% (320/509) of those clustered phylogenetically. Of the 156 named partners of these 509, 76% (118/156) clustered phylogenetically. Cluster sizes ranged from 2-31 (mean = 3.4), of which a mean of 0.20 partners were named in the CTDB.

**Conclusion:**

Integration between molecular epidemiology and contact tracing data may be synergistic to disrupt HIV transmission given the only moderate concordance between them. The existing concordance between the databases allows better characterization of the local HIV epidemic, while the discordance suggests data incompleteness in both databases, informing public health towards investigations to disrupt transmission.

**Disclosures:**

**All Authors**: No reported disclosures

